# Country Differences in Financial Preparation: What Do People Expect From the State?

**DOI:** 10.1093/geroni/igab046.728

**Published:** 2021-12-17

**Authors:** Jana Nikitin, Maria Clara P Paula de Couto, Sylvie Graf, Klaus Rothermund

**Affiliations:** 1 University of Vienna, Vienna, Wien, Austria; 2 Friedrich-Schiller University Jena, Jena, Thuringen, Germany; 3 University of Bern, Bern, Bern, Switzerland

## Abstract

A considerable gap between one’s pension and living expenses in old age exists in almost all developed countries, making savings and financial preparation for old age inevitable. Nevertheless, financial preparation for old age substantially differs across countries. Using the data from the AAF project, we investigated what motivates people in different countries (USA, Germany, Czech Republic, Hong Kong, and Taiwan) to financially prepare for old age. Financial preparation was the highest in the USA, followed by Germany and the Czech Republic. The lowest levels of financial preparation were found in Hong Kong and Taiwan. These differences were explained by age-related expectations on a “paternalistic” role of the state: Greater endorsement of the idea that the state should provide financial support to older citizens led to less preparation. These findings are in line with the idea that individuals’ beliefs and expectations regarding the role of institutions shape personal actions.

